# Leave No Photon Behind: Artificial Intelligence in Multiscale Physics of Photocatalyst and Photoreactor Design

**DOI:** 10.1002/advs.202306604

**Published:** 2024-03-13

**Authors:** Joel Yi Yang Loh, Andrew Wang, Abhinav Mohan, Athanasios A. Tountas, Abdelaziz M. Gouda, Alexandra Tavasoli, Geoffrey A. Ozin

**Affiliations:** ^1^ Solar Fuels Group, Department of Chemistry University of Toronto 80 St. George Street Toronto Ontario M5S 3H6 Canada; ^2^ The Department of Electrical and Electronic Engineering The Photon Science Institute Alan Turing Building, Oxford Rd Manchester M13 9PY UK; ^3^ The Department of Chemical Engineering and Applied Chemistry 200 College St, Toronto Ontario M5S 3E5 Canada; ^4^ The Department of Mechanical Engineering University of British Columbia 6250 Applied Science Ln #2054 Vancouver BC V6T 1Z4 Canada

**Keywords:** carbon dioxide reduction, fischer tropsch, material modeling, neural network, porous supports, photoreactor modeling

## Abstract

Although solar fuels photocatalysis offers the promise of converting carbon dioxide directly with sunlight as commercially scalable solutions have remained elusive over the past few decades, despite significant advancements in photocatalysis band‐gap engineering and atomic site activity. The primary challenge lies not in the discovery of new catalyst materials, which are abundant, but in overcoming the bottlenecks related to material‐photoreactor synergy. These factors include achieving photogeneration and charge‐carrier recombination at reactive sites, utilizing high mass transfer efficiency supports, maximizing solar collection, and achieving uniform light distribution within a reactor. Addressing this multi‐dimensional problem necessitates harnessing machine learning techniques to analyze real‐world data from photoreactors and material properties. In this perspective, the challenges are outlined associated with each bottleneck factor, review relevant data analysis studies, and assess the requirements for developing a comprehensive solution that can unlock the full potential of solar fuels photocatalysis technology. Physics‐informed machine learning (or Physics Neural Networks) may be the key to advancing this important area from disparate data towards optimal reactor solutions.

## Graphical abstract

Recent strides in Language Neural Networks, like Generative Pre‐Trained Transformer (GPT), offer renewed optimism in achieving General Artificial Intelligence. This prompts a re‐evaluation of previously unsolvable problems due to scale and complexity. Four pivotal design factors are distill of a CO2‐to‐solar fuel plant, from solar photon to carbon‐products, and discuss how machine learning can be used to maximize photonic efficiency.

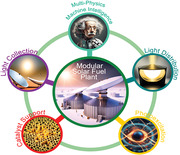

## 1 Introduction

Solar and wind energy play crucial roles in transitioning our society away from fossil energy, yet the demand for fossil fuels continues to rise, leading to increased greenhouse gas emissions globally. To address this paradox, efforts have been made to capture, store, and utilize excess carbon dioxide from the atmosphere. One approach is to recycle captured CO_2_ emissions to produce sustainable chemicals and fuels. While high‐performance CO_2_ photocatalysts have been discovered, the main challenge lies in the overall solar‐to‐product efficiency, which involves integrating photocatalyst and photoreactor materials, chemistry, and optics. Currently, many of these components have been optimized separately with various figures of merits, without consideration of optimizing the operation of two or more of these components together.

**Figure 1:**
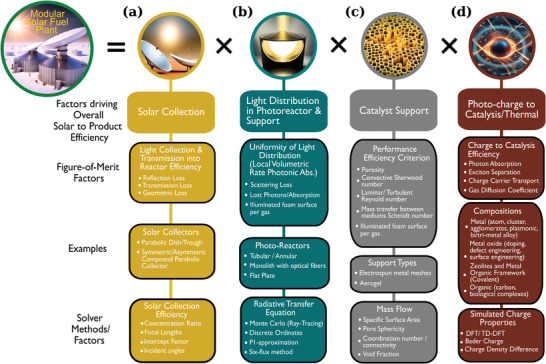
A practical modular CO_2_ recycling plant energized by sunlight will require at least four collector‐reactor‐support‐material design factors to be optimized by machine learning methods. Several fundamental factors associated with theoretical figure of merits are shown in the second row from top. Some examples investigated in the literature, and solver method or solver variables shown in the remaining two rows.

In this discussion, we focus on a hypothetical gas‐CO_2_ reducing/reforming modular solar‐photoreactor consisting of nano‐micron sized powder agglomerates of a photoactive catalyst loaded on a scaffold support suitable for light penetration. For such a solar‐photoreactor system, several figures of merit have to be considered (**Figure** **1**). The first factor is the solar collection efficiency (Figure 1a), where various losses reduce the amount of light transmitted into the reactor. The second factor is the local volumetric rate of photon absorption within the photoreactor (Figure 1b), which is complicated by the geometry of the reactor and the catalyst material on the support. Ideally, the light distribution within the reactor and support should be uniform such that the local rate of photon absorption is similar across all available surface of the catalyst. The third factor is associated with the support (Figure 1c), which has to maximize mass transfer efficiency while also maximizing the illumination per surface area of the catalytic material as light penetrates the support structure. Finally, after accounting for the photoabsorption coefficient of the catalytic material, it is necessary to consider the photogeneration of charge carriers that generate a surface charge imbalance for the reaction to proceed (Figure 1d). The effectiveness of the surface charge is modulated by carrier transport properties and reactant gas diffusion on the surface. All in all, after a tortuous path, a photon that enters the solar collection optic is converted to an effective charge that converts reactants to product molecules.

In each section, we explore the fundamental variables that underpin the figures of merit of these four stages and highlight the role of machine learning in advancing the optimization of the reactor, support, and materials. We will briefly discuss several examples that were evaluated by these figures of merit. In the last section, we will explore the potential use of physics‐based neural networks to synthesize these variables into a model for design simulations.

## 2 Light Collection Optics

The first component in the photoreactor system is the light collection system, also called collection optics, which serves the purpose of concentrating photons from a light source (i.e., the sun or electrically driven lamps) towards or onto a targeted area on the photoreactor, which may be a window, glass tubing, or some other structure.^[^ ^1^ ^]^ The photoreactor then has its own optimized geometry that facilitates the efficient delivery of photons from the window of the reactor to the surface of the photocatalyst, as will be discussed later on in this article.

There are two characteristics of photon flux that should be considered when designing collection optics. The first is the rate of photon flux (the amount of energy per time) that falls into the photoreactor. The second is illumination, which is the degree of homogeneity in the spatial distribution of the photon flux on the targeted area of the photoreactor, specifically whether it becomes evenly spread out over the desired target area^[^ ^2^ ^]^ such as the catalytic‐material‐coated support. For this purpose, non‐imaging optics are most commonly used to facilitate the transfer of photons from the sun to the photoreactor target. For applications that use sunlight, non‐imaging collectors can be stationary or can use solar tracking. As sunlight is collected by this optical system, it is typically concentrated through a viewing port on the photoreactor. The concentration ratio (*C)* for geometric optics is defined as $C_{\mathrm{geometric}}=\frac{A_{1}}{A_{2}}$, where *A_1_* and *A_2_* represent the radiation incident on the aperture area and the radiation incident on the absorber (photoreactor target), respectively. There is a thermodynamic limit to geometric concentration,^[^ ^3^ ^]^ given by $C_{\max}=\frac{n}{\sin\theta }$ or $C_{\max}=\frac{2n}{\sin2\theta }$ depending on the geometry at hand, where *n* is the index of refraction at the absorber surface, likely the photoreactor window, and θ is the half angle of acceptance. A system that can obtain *C*_max_ is considered “ideal”. Completely stationary concentrators can deliver moderate levels of concentration, from 1 to 4×, whereas higher concentrations from 10 to 40 000× require tracking. Examples have been shown that concentrate sunlight up to 80 000×.^[^ ^4^ ^]^

**Figure 2:**
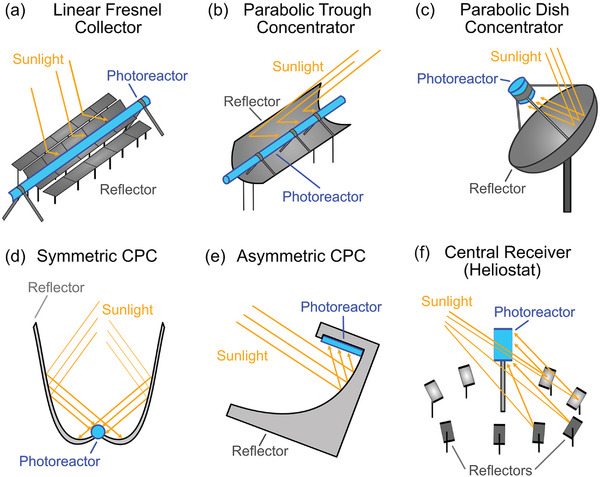
a) Linear Fresnel collectors are parallel arrays of flat tilted mirror segments that focus sunlight onto a photoreactor absorbing tube above the mirror field. Flat mirrors are inexpensive but distribute sunlight over a large area. b) Parabolic trough concentrators have a higher optical efficiency, as the curvature focuses sunlight onto a single focal line. c) Parabolic dish concentrators have the focal point as single point, enabling extremely high optical efficiencies. d) A parabolic concentrator is optimized to collect light at a specific angle, whereas compound parabolic collectors (CPCs) comprise of multiple parabolic segments optimized for a large range of incoming light angles. This reduces the need for a solar tracker. The photoreactor is a tube absorber. e) Asymmetric compound parabolic collectors are more suitable for collecting non‐uniform or off‐normal axis incoming light. f) Heliostats can direct sunlight to a central receiver like a tower‐based photoreactor. Heliostat arrays can cover a larger area than the collection area of an array of parabolic dish concentrators.

The type of non‐imaging collectors used for this purpose include symmetric and asymmetric compound parabolic concentrators (CPCs), parabolic dish concentrators, central receivers (heliostats), parabolic trough concentrators, and linear Fresnel collectors.^[^ ^5^ ^]^ Simplified schematics of these devices are depicted in **Figure** **2**. CPCs are, to date, the most promising geometric optic collectors for solar energy applications. Compound parabolic concentrators (CPCs) consist of a trough with two sections of a parabola facing each other. They make use of multiple internal reflections to direct any radiation to the target that is at the bottom of the reflector, and they may be symmetrically or asymmetrically designed, depending on the photoreactor geometry and structural constraints of the system design. Parabolic dish collectors use a mirrored curved dish that directs and concentrates sunlight onto a photoreactor target. Central receivers, also called “power towers” or heliostats, consist of a large field of flat, sun‐tracking mirrors called heliostats that focus and concentrate sunlight onto the top of a receiver tower. Parabolic trough systems^[^ ^6^ ^]^ put photoreactors along the focal line of each parabolic mirror and are the most mature technology for solar concentration that can facilitate roughly one‐third of the theoretical maximum concentration. Linear Fresnel collector systems position the photoreactor above several mirrors, allowing the mirrors greater mobility when tracking the sun. Linear Fresnel collectors can concentrate light up to 30×.

If the objective is to maximize the photon flux at the photoreactor target, then the optical efficiency of the collection system can be rationalized as the proportion of incident photon flux on the concentrator that is delivered to the target. In this case, the efficiency of the collection system can be represented by $\eta_{C}=\phi_{PR}/\phi_{0}$, where *η_C_* is the efficiency of the light collection stage, ϕ_*PR*_ is the photon flux at the viewing port of the photoreactor, and ϕ_0_ is the photon flux that is originally incident on the collection system.

Losses can limit optical efficiency of these collectors. The first type of loss is geometric loss, which includes physical shading or blocking of the absorber or reflectors by mechanical components or external physicality. The second type is reflection or transmission losses that stem from non‐idealities in the construction materials used to build the collection optics system. The third type is transmission losses that occur when light passes through materials like the reactor housing or window. Reflection and transmission losses are accounted for in the overall efficiency calculation of the system, which is defined as the ratio of the number of photons “used” by the catalytic system to the number of photons incident on the system. The photons can be “used” through either photochemical or photothermal routes to drive the reaction. Reflection losses can happen at both the concentrator and the photoreactor window, whereas transmission losses occur only when the photons pass through the photoreactor window and are a function of the photon energy, angle of incidence, thickness of the window, and the construction material of the window. Nonetheless, after considering these losses, in comparison to imaging collectors, non‐imaging collectors can achieve the highest possible levels of concentration with the most relaxed optical defect tolerances and can achieve uniform illumination within the photoreactor.^[^ ^7^, ^8^ ^]^ However, non‐imaging collectors have the disadvantage that they typically have a reflecting surface made up of multiple components, which results in optical losses.^[^ ^9^ ^]^

Artificial intelligence (AI) and machine learning methods have been used in a number of ways to improve the performance of light collection systems. For example, they have been used to predict the optical efficiency of concentration systems so as to modify their geometric design to achieve an optimized efficiency.^[^ ^10^, ^11^ ^]^ A multivariate loop optimization was used to derive the optical efficiency equation based on geometric parameters such as the concentration ratio, focal lengths, and incident angles of illumination on a parabolic trough collector. With this equation, that is also validated by ray‐tracing software, a concentrator based on a secondary reflection hyperbola surface was designed to achieve a high effective geometric concentration ratio. In addition, machine learning methods have also been applied to predict solar insolation in order to forecast the energy that will be available.^[^ ^12^, ^13^ ^]^

## 3 Light Distribution

Before discussing the approach used to determine the light distribution within a photoreactor, we will briefly discuss light distribution vis‐à‐vis the advantages and disadvantages of various photoreactor designs^[^ ^14^ ^]^ such as the annular/tubular reactor,^[^ ^15^, ^16^ ^]^ planar microreactor,^[^ ^17^, ^18^ ^]^ and honeycomb monolithic reactor. In tubular reactors, one or more tubular lamps are typically placed in the center of a cylindrical vessel, often enclosed in quartz sleeves to isolate them from the reacting medium. The intensity of radiation from these lamps decreases as the distance from the source increases, which can be partially negated with the reactor walls acting as reflectors. The non‐uniformity of light distribution is further increased by having a catalyst‐coated foam^[^ ^19^ ^]^ around the lamp. The advantages of monolithic photoreactors are their compactness, high flow rates at low‐pressure drops and their high surface‐to‐volume ratios.^[^ ^20^, ^21^, ^22^ ^]^ However, the fast decay of light from the front of the honeycomb necessitates internal illumination typically provided by optical fibers. Such an internal illumination has been shown to effectively increase reaction photoefficiency from 22% to 96.5% in gas‐phase xylene degradation,^[^ ^23^ ^]^ at the expense of a lower volume of catalyst within the reactor. A simpler photoreactor configuration is the planar microreactor^[^ ^24^, ^25^, ^26^ ^]^ with a wide and shallow channel that covers a wide planar substrate. Similar to the monolithic reactor, the amount of catalyst is limited, which has encouraged investigations of various LED light sources^[^ ^27^ ^]^ for uniform irradiance on the surface of the catalyst and optical output stability with a minimal number of light sources. As the distance between the LED and the microreactor channel increases, light distribution uniformity across the channel surface improves due to greater light dispersion and blending. However, this also reduces the photon flux reaching the microreactor surface because of the increased light dispersion.

As was confirmed in the aforementioned investigations, generally, the ideal light distribution within a photoreactor is uniform,^[^ ^28^ ^]^ which results in a uniform local volumetric rate of photon absorption (LVRPA). This in turn maximizes the efficiency of the photocatalytic kinetics due to the minimization of the recombination rate of the electron‐hole pairs.^[^ ^29^ ^]^ However, the light that will be entering a solar fuel photoreactor will likely be intensified by a solar collector, which will result in an incident flux through the reactor window at multiple angles. Considering that the presence of the support‐catalyst structures and the reactor walls or gas ports will also result in scattering, designing a reactor with a uniform light absorption is not easily achieved without solving the Radiative Transport Equation, (RTE).^[^ ^30^, ^31^ ^]^ The expression of the RTE is:

(1)

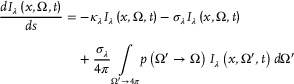




**Figure 3:**
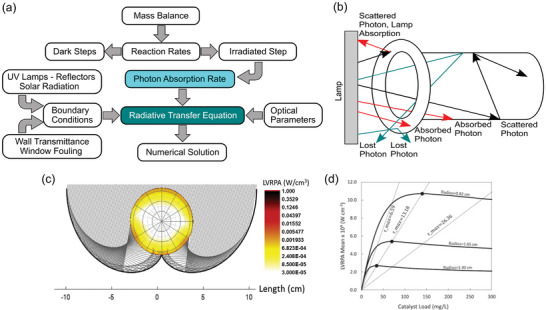
a) Solving the Radiative Transfer Equation (RTE) for a specific reactor geometry and scattering objects results in a distribution of the photon absorption rate. Adapted from. ^[^ ^31^ ^]^ b) Types of photon fates for an uncollimated light source shining into a monolith reactor for a Monte Carlo‐based solution of the RTE. Adapted from. ^[^ ^32^ ^]^ c) Ray tracing and six‐flux method employed in a compound parabolic collector with a TiO2 particle absorber in the middle. The calculated LVRPA for an absorber of 2.5 cm radius shows some absorption beyond the boundary layer. d) The LVRPA for different absorber radii as a function of catalyst load. The dotted line indicates the effective optical constant considering an optimal diameter of various TiO_2_ polymorphs (Degussa with a τ of 13.38). Adapted from. ^[^ ^33^ ^]^

The first term on the right‐hand side of the equation is the absorption of the incident light, the second term is the out‐scattering with an attenuation of the specific intensity at the wavelength, and the third term is the contribution of scattered radiation to the specific intensity at the wavelength, where the monochromatic radiation intensity is a function of position x, direction Ω, and time t. The *I*_λ_(*x*,Ω, *t*) is the specific spectral intensity for characterizing the radiation field in a photochemical reactor, Einstein per square meter per steradian per nm per second. S is the distance traveled by radiation through that medium. *p*(Ω′ → Ω) is the phase function, which describes the probability that a photon is scattered at an angle relative to its original direction. The scattering term is integrated over the solid angle (ratio of the surface area of the object to the square of its distance from observer); σ_λ_ describes the probability of a photon being scattered (per unit distance). The RTE is assumed to be valid in a homogenous medium with negligible emission radiation from the heating of the catalyst + support. There is one boundary condition at the surface of the entrance. The port and the reflective surfaces define the directions that effectively contribute to the reactor space radiation field (**Figure** **3**).

There are several models for determining the radiation field distribution in photoreactors: the Monte Carlo method,^[^ ^32^, ^34^ ^]^ P1 approximation method,^[^ ^35^, ^36^ ^]^ discrete ordinates method,^[^ ^37^, ^38^ ^]^ and six‐flux method.^[^ ^33^ ^]^ The Monte Carlo method is a stochastic method that simulates the behavior of photons in the reactor by following the probable path of discrete bundles of photons until their final fate (absorption or escape from the reactor) is established. The Monte Carlo method has been successfully used for multiple reactor geometries and can be employed for reactors of complex geometries. The discrete ordinates method,^[^ ^39^ ^]^ on the other hand, is a deterministic method that solves the RTE by dividing the radiation field into discrete volumes and solving the RTE for each volume. This method is based on approximating the angular distribution of the radiation intensity and can be used to determine the radiation field in the reactor. The discrete ordinates method is more computationally efficient than the Monte Carlo method but may not be as accurate for complex geometries. The P1 approximation method simplifies the RTE by assuming that the radiation field can be represented by a linear combination of the radiation energy density and the vector intensity encompassing the direction and polarization. The radiative flux is expanded in terms of spherical harmonics, and only the first‐order expansion term (P1) is retained. However, the method assumes that the angular distribution of radiation intensity is isotropic and is thus limited to symmetrical reactor shapes. The six‐flux method^[^ ^33^ ^]^ is used to solve the RTE by simplifying the number of independent variables. This is achieved by balancing the differential element for the incident radiant energy *G*_λ_(*x*,*t*) with the spectral irradiance *I*_λ,Ω_(*x*, *t*). The LVRPA is related to the incident G by:

(2) $$\int_{\Omega }I_{\lambda ,\Omega }\left(x,t\right)d\Omega =G_{\lambda }\left(x,t\right)$$

(3) $$\mathrm{LVRPA}\left(x,t\right)=\kappa \left(x,t\right)G\left(x,t\right)$$

The photons are assumed to scatter in only six Cartesian directions according to a probabilistic scattering function, thus eliminating directional coordinates.

A commonly used figure of merit for the efficiency of the photocatalytic process in a reactor is the Local Volume Rate of Photon Absorption (LVRPA), which is linked to the solution of the RTE equation.

Considering the complexity of optimizing different shapes and sizes in photoreactors, machine learning has been applied to overcome challenges in video rendering of lighting environments similar to those applicable in the light distribution within photoreactors. The challenge of rendering complex, high‐quality photorealistic scenes with multiple light sources, reflections, and shadows requires significant computational resources under conventional rendering techniques such as ray tracing. Thus, there are multiple neural network approaches that seek to accelerate the rendering process and attain amore accurate image of the radiance distribution field. There are several methods for predicting the radiation field distribution, including Deep Radiance Caching^[^ ^40^ ^]^ and Neural Ray Tracing.^[^ ^41^ ^]^ Ray tracing proceeds by tracing the path of light rays from a camera through each pixel of an image plane as they interact with the objects in the scene. The interaction of reflection, refraction, and absorption are calculated. Neural ray tracing trains a neural network to predict the lighting scene, given the camera position and material properties of the objects in the scene. Neural ray tracing can thus reduce the number of ray‐tracing calculations and be more flexible, as the neural network can be trained on a wide range of input parameters, at the cost of accuracy. Inaccuracies from the entanglement of surface modeling, light effects, and scattering distribution functions can be mitigated by decomposing the light transport and training separate neural networks for each component. Similar to neural ray tracing, neural cache radiance involves initial training on a set of images of the scene followed by caching the radiance values at certain points in the scene. The cache can then be used to interpolate the radiance values at other points in the scene, reducing the number of expensive ray‐tracing computations required.

## 4 Support Characteristics

**Figure 4:**
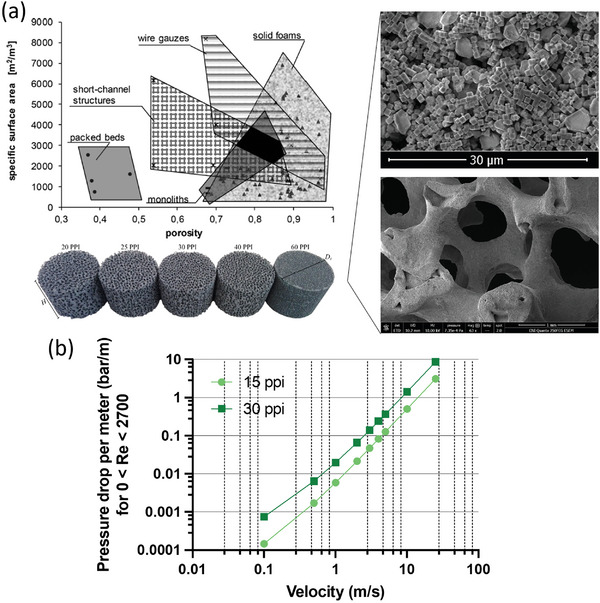
a) top left: Comparison of different catalyst supports,^[^ ^47^ ^]^ bottom left: Examples of solid SiC ceramic foam porosities,^[^ ^52^ ^]^ bottom right: Photocatalyst‐loaded alumina ceramic support (30 ppi) imaged using ESEM, top right: Magnified photocatalyst consisting of cubic Cu_2_O (≈1 micron edge) loaded on a 30 ppi alumina ceramic foam support.^[^ ^53^ ^]^ b) Pressure drop per meter versus velocity past two different porosity alumina foams^[^ ^43^, ^47^ ^]^ in the laminar flow regime 0 < *Re* < 2700.

As the transmitted sunlight is distributed within a photoreactor, some of it will be incident on and penetrate the catalyst‐coated support, which in turn has various advantages. A number of studies have indeed shown that loading photocatalysts on nano/meso/macro‐porous supports enhances their activity, as their porosity is increased due to light distribution into the supports.^[^ ^42^, ^43^ ^]^ Furthermore, these systems have been shown to outperform slurry‐based systems^[^ ^42^ ^]^ used frequently for aqueous organic decomposition, while avoiding the need for costly separation steps.^[^ ^44^ ^]^ Different types of supports have been investigated for intensifying thermal catalysis, as shown in **Figure** **4**, and are now being applied to photocatalysis. For example, nanoscale pores on the order of ≈30 nm found in aerogels^[^ ^45^ ^]^ show promise, as the proximity of struts in the structure enhances light scattering; however, limitations in transport properties occur whereby the nano pores cause slow diffusion into and out of the structure, resulting in high contact time (and potentially back reactions) which may limit their effectiveness for fast reaction processes. In that report,^[^ ^45^ ^]^ an aerogel support offered about a ≈twofold enhancement in the absorption rate compared to packed particles when both were illuminated from top and bottom, the benefits likely limited by poor absorption and back reflection losses. Similarly, mesopores with pore sizes on the order of microns were found to allow up to tenfold the light penetration compared to powders^[^ ^46^ ^]^; however, high pressure drop for these pores sizes remains limiting. Finally, macropore systems with approximately mm‐size pores (see Figure 4a) made from materials as diverse as metals and alloys, ceramics, polymers, glass, or carbon,^[^ ^47^ ^]^ strike a balance between high light penetration and medium to high‐throughput transport (heat/mass/flow) characteristics.^[^ ^42^, ^43^ ^]^ They can be illuminated from one^[^ ^48^ ^]^ or multiple angles depending on the reactor design. However, the larger the pore size, the lower the crush strength,^[^ ^49^ ^]^ which is an important consideration when planning for commercial, high‐throughput systems that exhibit non‐negligible pressure drop as shown in Figure 4b.

Many photocatalyst studies on powders have demonstrated that the reaction rate scales linearly with light intensity. This can be understood to be due to a scalar increase in the number of photons on a constant active surface area, increasing the activity (assuming the higher intensity does not lead to faster electron‐hole pair recombination). Films that are amenable to light intensification have been studied that can cover a large surface area; however, their footprint area is typically restrictive.^[^ ^45^, ^50^ ^]^ Therefore, the challenges that photocatalyst supports attempt to address when planning for photocatalytic process intensification are optimizing both the photon and transport characteristics. For the former, a metric used to quantify the extent of photon transfer or illumination efficiency of the light‐harvesting capabilities of a given support can be expressed in terms of a “photonic” or non‐absorbed light coverage, as given by Van Gerven et al.^[^ ^51^ ^]^:

(4) $$\eta_{\mathrm{ill}}=\kappa \cdot \left(\frac{P_{\mathrm{cat}}}{P_{\mathrm{lamp}}}\right)\cdot \left(\frac{A_{\min E}}{A_{\mathrm{cat}}}\right)$$

where η_ill_ is the illumination efficiency (m^−1^ or %), κ the illuminated surface per unit of internal gas or liquid volume inside the reactor (m_ill_^2^   m_reactor_^−3^ or m^−1^), P_cat_ the radiant power incident on the catalyst or support surface (W), P_lamp_ the radiant power emitted from the lamp or light source (W), A_min E_ the catalyst or support surface area that receives at least the band‐gap energy (m^2^), and A_cat_ the total catalyst or support surface (m^2^). Having a highly illuminated catalyst surface area with photon energy above the band gap needed for photoexcitation (or a prolonged photon mean‐free path) per volume of support allows for effective contact between photons and the photocatalyst.^[^ ^51^ ^]^

In terms of maximizing transport properties, a support needs to be robust enough to allow moderate to high Reynolds/Nusselt/Sherwood numbers and associated effective flow/heat/mass transport. This is to ensure that the boundary layer thicknesses remain negligible enough to facilitate low pumping (operating costs), rapid heat transfer (avoiding hot/cool spots), and rapid replenishment/removal of reactants/products for reaction rate control. A figure of merit for process transport intensification is the support's performance efficiency criterion (PEC).^[^ ^47^ ^]^ This uses the ratio of the mass transfer resistance to flow resistance.

(5) $$\mathrm{PEC}=\frac{\ln\left(\frac{C_{A0}}{C_{\mathrm{AL}}}\right)}{\left(\frac{\Delta P}{\rho w^{2}}\right)}=\left(\frac{\frac{k_{c}\cdot S_{v}}{\left(1+k_{c}/k_{r}\right)}\cdot \frac{L}{w}}{2\cdot f\cdot \left(\frac{L}{D_{h}}\right)}\right)=\left(\frac{D_{h}\cdot k_{c}\cdot S_{v}}{2\cdot w\cdot f\cdot \left(1+\frac{k_{c}}{k_{r}}\right)}\right)$$

where C_A_ (molm^−3^) is at the inlet (C_A0_) or outlet (C_AL_), ΔP is the pressure drop (Pa), ρ the fluid density (kgm^−3^), k_c_ the mass transfer coefficient (ms‐^1^), k_r_ the reaction rate constant, S_v_ the specific surface area (m^2^m^−3^), L the bed length (m), w the superficial velocity (ms^−1^), f the Fanning friction factor, and D_h_ the hydraulic diameter. Rewriting this for the mass transfer control condition k_r_ ≫ k_c_, we can write:

(6) PEC=−ln1−XΔPρw2=Dh·kc·Sv2·w·f·Re·Sc=Dh·Sv·Sh·ε2·f·Re·Sc

where *Sh* is the Sherwood number, *ε* the porosity (based on the Reynolds number definition), *Re* the Reynolds number, and *Sc* the Schmidt number (ratio of momentum diffusivity to mass diffusivity).

Generally, this criterion is the ratio of reactor yield to flow resistance, and the higher the value the better (high yield, low operating cost). Packed beds typically have lower PEC values due to the high pressure drop experienced, whereas monoliths typically have higher values constant with *Re*. Wire gauzes have PEC maxima exceeding those of monoliths for some *Re* ranges (e.g., 50–1000), while short‐channeled structures have lower PECs than those of monoliths and wire gauzes, but their PECs improve with increasing *Re*. Solid foams^[^ ^52^ ^]^ also display PEC maxima which exceed those of short‐channeled supports for low to moderate *Re* ranges (e.g., 100–500) but deteriorate as the *Re* number increases. The reader is referred to^[^ ^47^ ^]^ for a more detailed discussion. Looking at flow resistance of solid foams, Figure 4b displays a remarkably favorable pressure drop of ≈1 bar m^−1^ at 10 m s^−1^ superficial velocity; however, this is for the best‐case laminar flow region.^[^ ^47^ ^]^ An example of a support that integrates photon and transport properties is the optical fiber monolith reactor, which combines a classic monolithic structure (with high and constant PEC) with many parallel channels with high‐surface‐area light utilization, whereby it can attain tenfold the illumination efficiency compared to annular reactors.^[^ ^42^, ^50^ ^]^ The authors are developing ways of finding porous support “light fingerprints” as a function of the absorption coefficient that look at the isophotonicity of the supports and ways to optimize their intrinsic light‐capturing properties. Preliminary data suggests that smaller pore foams capture light in their inner volumes better than larger pore foams, with both losing photon absorption capacity rapidly towards their exteriors. A modified PEC based on photon transfer over pressure drop is proposed, where a higher value of the PEC is better for a foam's light utilization from a pumping energy standpoint:

(7) $$\mathrm{PEC}=\left(\frac{N_{\mathrm{PA}}}{N_{P0}}\right)/\left(\frac{\Delta P}{\rho w^{2}}\right)$$

where N_PA_ is the photon absorbed, and N_P0_ is the photon incident.

We will now discuss the application of machine learning (ML) to developing porous materials. While an investigation of both illumination and mass transport in porous materials has not been done before to our knowledge, there are some examples related to optimizing mass transfer. One well‐studied class of materials is metal‐organic frameworks (MOFs) that have applications ranging from gas separation, storage, water remediation, and supercapacitors to catalysis. MOFs are 2D or 3D frameworks composed of repeating metal and organic ligands that form a well‐ordered network containing voids. Some identified MOFs with bulk sizes on the order of sugar cubes can exhibit surface areas the equivalent of 6 football fields.^[^ ^54^ ^]^ One research group trained several ML models on 10^4^ MOFs using the geometric features of pore size, surface area, and void fraction to test a set of ≈10^5^ MOFs for methane storage capacities. They were able to identify the minimum MOF densities and void fractions needed to optimize storage as a function of system pressure.^[^ ^55^ ^]^

Another application of ML to developing porous structures is to predict reaction rates of chemical reactions through porous media as a function of a few important structural features. Liu et al.^[^ ^56^ ^]^ identified and ranked 11 porous geometry features, out of which specific surface area effect (ratio of total surface area to bulk volume), pore sphericity shape effect (describing the smoothness of the reactive surface affecting mass and fluid transfer), and coordination number flow/connectivity effect (the average number of throats connected to a pore or the average connectivity of the pore space) were the most important. They mention, for example, that the framework could be extended to optimize mechanical strength as well, a limitation of increasingly open pore foams. Naturally, ML could be extended to optimizing porous support materials for photochemistry by identifying key parameters of the parameter space including those in the illumination efficiency equation (Equation 4, illuminated area, light properties (intensity, wavelength, polarization, etc.), attenuation coefficient, or effective illuminated area along with the 3 important structural features identified by Lui and colleagues.

## 5 Photoexcitation Efficiency in Material

As light is incident on the photocatalytic material, it can drive electronic and thermal behavior. We thus take a step back to discuss how the improvement of photocatalyst efficiency through design can be approached by delineating specific parameters and how they are related to each of the process efficiencies that make up photocatalysis with a consideration of the length and time scale window in which both light‐matter interactions and the surface reaction are relevant. Productive photon‐to‐chemical transformation can proceed through dominantly photochemical, dominantly photothermal, or synergistically photo‐thermal‐chemical routes depending on the extent of photoexcitation, charge carrier participation, recombination, and heat transfer. The overall photocatalytic efficiency^[^ ^57^ ^]^ can thus be expressed as a sum of the photothermal and photochemical efficiencies (related to the quantum efficiency (QE)) (Equation 8). Photochemical efficiency is a multiplication of the fundamental process efficiencies: photon absorption, exciton separation, carrier diffusion, and transport and mass transfer (Equation 9)

**Figure 5:**
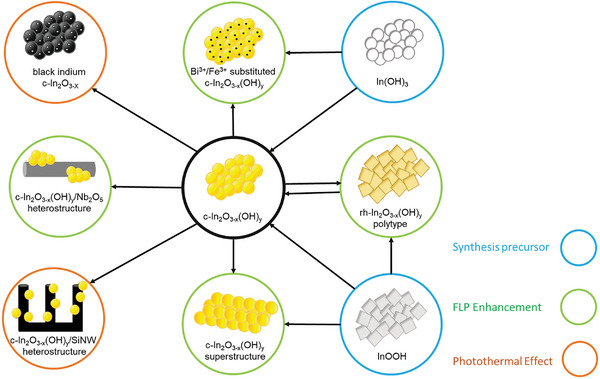
Synthetic pathways and relationships between different forms of defected indium oxide. Adapted with permission.^[^ ^132^ ^]^

**Figure 6:**
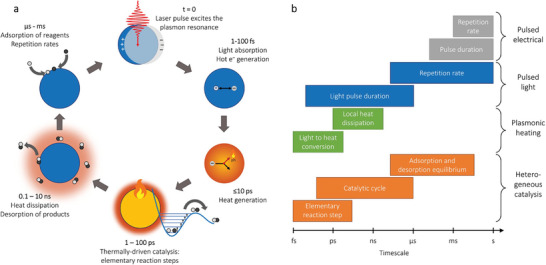
Timescales involved with different processes listed in Table 1. Adapted with permission.^[^ ^133^ ^]^

There are several classes of photocatalytic materials (metal oxides, metals, carbon and graphitic carbon nitride, perovskites, and metal organic frameworks capable of CO_2_ reduction).^[^ ^57^ ^]^ As shown in **Figure** **5**, there are several modifications that can be made to a given catalyst to improve the individual process efficiencies (**Figure** **6**).

(8) $$\eta_{\mathrm{photocatalysis}}=\eta_{\mathrm{photothermal}}+\eta_{\mathrm{photochemical}}$$

(9) $$\begin{array}{cc} \eta_{\mathrm{photochemical}} & =\eta_{\mathrm{light}\mathrm{absorption}}\times \eta_{\mathrm{exciton}\mathrm{seperation}}\\ & \;\times \eta_{\mathrm{carrier}\mathrm{diffusion}-\mathrm{transport}}\times \eta_{\mathrm{mass}\mathrm{transfer}} \end{array}$$

Given the myriad processes involved, **Table** **1** is presented as a summary with the key variables associated with each process and their fundamental relationship. All process efficiencies are dependent on the local temperature of the catalyst and electronic structure of the photocatalyst components, which comprise of surface and bulk density of states and the band gap.^[^ ^58^ ^]^

**Table 1:** A list of process efficiencies from a photothermal and photochemical perspective and the associated parameters/variables that affect the efficiencies.

Process Efficiency	Key Parameters/Variables	Constants	Formulaic Relationship
Photon Absorption	Refractive index Band gap Absorption coefficient, α(λ) Real, imaginary, and medium dielectric constant, ε_1_, ε_2_, and ε_*m*_ Transmittance, T Reflectance, R	None	$\sigma_{\mathrm{ext}}=\frac{\varepsilon_{2}}{{\left[\varepsilon_{1}+2\varepsilon_{m}\right]}^{2}+\varepsilon_{2}^{2}}$ (nanoparticle) $\alpha =\frac{1}{d}\ln\frac{T}{{\left(1-R\right)}^{2}}$ (film)
Exciton Separation (Photochemical‐Specific) *R_ex_*	Exciton binding energy, (the energy required to ionize an exciton from its lowest energy state), E_b_ Effective mass of exciton, m* Relative permittivity (dielectric constant), ε_*r*_	Elementary charge, *e* Planck's constant, *h*	$R_{\mathrm{ex}}=\frac{m^{*}e^{4}}{2h^{2}\varepsilon_{r}^{2}}=E_{1}\frac{m^{*}}{\varepsilon_{r}^{2}}$ (Eq. 3)
Charge carrier diffusion and transport, *J*	Carrier diffusion coefficient, *D* Carrier concentration, *n*, *p* Carrier mobility, µ Carrier lifetime, τ Electric field, E Collision time of the charge carrier, τ_*c*_ Defect type and concentration gradient ∇_*n*_ and ∇_*p*_	Boltzmann constant, *K_B_* Charge carrier effective mass, m* Elementary charge, *e*	*J* = *J_diffusion_* + *J_drift_* *J_n_* = *eD_n_* ∇_*n*_ + *ne*µ_*n*_ *E* (Equation 4.1) *J_p_* = − *eD_p_* ∇_*p*_ + *pe*µ_*p*_ *E* (Equation 4.2) Where $D=\frac{K_{B}T}{e}\mu$ and $\mu =e\frac{\tau_{c}}{m^{*}}$
Diffusion coefficient, D_G_ (Hirschfelder‐Bird‐Spotz)	Gas diffusion coefficient, *D_G_*	Collison integral, Ω Molecular weight of solute gas, molecular weight of solvent gas average collision diameter, M_1_ and M_2_	$D_{G}=1.86\times 10^{-3}\frac{T^{\frac{3}{2}}\sqrt{\frac{1}{M_{1}}+\frac{1}{M_{2}}}}{p\sigma^{2}\Omega }$

The photothermal efficiency is determined by the increase in local temperature of the active site and is simply quantified as the thermal contribution to the overall photocatalytic rate. The photon absorption efficiency and extent of non‐radiative recombination are in competition with the photochemical efficiency of semiconductor materials with increasing temperature.^[^ ^59^, ^60^ ^]^ For simple, bulk photocatalyst systems, a photothermal conversion coefficient, $\tilde{\alpha }$, can be determined experimentally by accurate measurement of the local temperature and also by calculating the photon absorption (Table 1) and the heat transfer from the absorbed power, based on the effective thermal conductivity and catalyst geometry (T_local_ = T_external_ + $I_{mathrminc}\tilde{\alpha }$). Plasmonic metals are an exception in some cases, in which hot carrier generation and photochemical enhancement increase with temperature. Metal nanoparticles such as Ag, Au, Al, and Cu can exhibit up to a tenfold improvement in absorption at a particular incident frequency that matches the local oscillation of plasma, resulting in local surface plasmon resonance (LSPR). LSPR can be used to generate heat, enhance the electromagnetic field around the nanoparticle to generate electron hole pairs more readily in the vicinity of this enhanced field, and generate energetic charge carriers that can participate in surface reactions by injecting directly into adsorbate orbitals or via a dielectric for specific bond‐activation and through non‐thermal vibrational activation of reactant molecules, (i.e., not from photothermal heating of electrons or phonons). Several detailed reviews discuss design strategies, including effects of size, shape, and hybrid structures.^[^ ^61^, ^62^ ^]^

We now turn to discuss the photoexcitation of charge carriers. Generally, these are associated with semiconductors or metal oxides/nitrides which may not have a suitable energy band gap for solar absorption. However, it is now well known that charge carrier absorption can be extended further into the visible wavelengths, where most of solar radiation is, through defect engineering or addition of a sensitizer to modify the electronic band structure. Electronic structure methods such as the time‐dependent density functional theory (TD‐DFT)^[^ ^63^, ^64^ ^]^ and the many‐body perturbation theory (i.e., GW/BSE) can be used to calculate the dielectric function and optical properties such as the refractive index dispersion and band structure, all of which depend on the types of defects and their concentrations in the material.^[^ ^65^ ^]^ Additionally, there are emerging methods of achieving broader absorption by modulating the geometries and dimensions of the catalyst. This enables control of refractive index dispersion to achieve near‐perfect, broadband absorption through metasurface engineering^[^ ^66^ ^]^ and alternating refractive indices in a periodic structure to enable the slow photon effect in photonic crystals and omnidirectional absorption.^[^ ^67^, ^68^, ^69^ ^]^

Beyond photoabsorption to excitons (bound state of an electron and hole coupled by electrostatic attraction), the separation of excitons into electron and hole charges is essential before the excitons recombine and dissipate as heat. Separated electrons and holes disturb the charge neutrality of surfaces, which provides additional potential energy for driving catalysis. Thus, the exciton binding energy, which represents the energy required to ionize an exciton from its lowest energy state, is considered as an important criterion in selecting a promising photocatalyst.^[^ ^70^ ^]^ The efficient separation of excitons requires that the binding energy be lower than the room temperature thermal energy value of 25 meV.^[^ ^71^ ^]^ As listed in Table 1 and Equation 3, the value of exciton binding is mainly dependent on the effective masses and the dielectric constant that is in turn determined by the curvature of the conduction and valence bands. Currently, density functional theory (DFT) calculation and machine learning (ML) approaches can estimate exciton binding energies and effective masses as well as different crystal orientations with high accuracy.^[^ ^72^, ^73^ ^]^ Typically, high distortion in the crystal structure under different excitations creates an anisotropic electronic field upon exciton generation, and this field assists charge separation. For instance, in tri‐halide perovskites, which is an example of a high dielectric constant material, a recent study reported that increasing the temperature decreases the binding energy due to spontaneous free‐carrier generation following light absorption.^[^ ^74^ ^]^

We highlight that there can be a complex relationship between charge separation and increased recombination rates due to high temperature. For example, a study showed that increasing the temperature of the Fe/MoS_2_ catalyst and the adsorption of N_2_ prolonged the exciton lifetime of the catalyst and in turn increased its catalytic activity by suppressing the electron‐hole pair recombination in MoS_2_.^[^ ^75^ ^]^ Doping MoS_2_ with Fe prolongs the recombination time, suggesting that the metal exerts an enhanced degree of charge separation by accepting excited electrons. Another study revealed that increasing the temperature over TiO^2^ increased the lifetime of the photogenerated carriers, thus enhancing the H_2_ production.^[^ ^76^ ^]^ Glycerol was added to TiO_2_ as a hole scavenger to remove the photogenerated holes in the TiO_2_. This, in turn, gave a longer lifetime for the photogenerated electrons, especially at higher temperatures. However, elevating the temperature to 125 °C over Pt/g‐C_3_N_4_ catalyst and increasing the photoconductivity revealed an increased number of active charge carriers, while further heating to 150 °C promoted charge recombination and thus lower photoconductivity.^[^ ^77^ ^]^ Thus, optimizing the operating temperature in photochemical catalysis systems is crucial for balancing charge separation, minimizing recombination, and maximizing catalytic effectiveness.

Following charge separation, charge carrier diffusion towards the active site either via the surface or from bulk to surface is an effective modulator of the efficiency of the charge separation and drives the geometry of the catalysts in order to minimize recombination during transport. In principle, carrier diffusion and transport can be described in terms of electron/hole flow, which is driven by the concentration gradient and drift driven by the potential gradient across the photocatalytic system.^[^ ^78^ ^]^

The diffusion of molecules to the surface of the catalyst where adsorption and desorption steps can take place depends on surface structure, defect‐type, and concentration, which are temperature‐dependent.^[^ ^79^, ^80^ ^]^ Pulsed wave catalysis can leverage the ability of light to change the active site temperature to match the time scale of elementary steps, desorption, and adsorption events (which are on the order of picoseconds to microseconds).^[^ ^79^ ^]^ Spectroscopic approaches are being used to establish spectrum‐property relationships for adsorption energies.^[^ ^81^ ^]^

When addressing the efficiency of the surface reaction, difficulty in accurate quantification of an active site and photoactive centers makes it challenging to use universal metrics for catalyst efficiency like turnover frequency or turnover number, which are ubiquitous in homogenous catalysis as metrics for overall efficiency.^[^ ^82^ ^]^ Excluding absorption, all mentioned steps can be considered as rate‐determining step and are pertinent to be investigated for each material under the reaction conditions (determining which step is the most dominant one for the QE). Time‐resolved photoluminescence, transient absorption spectroscopy, and electrochemical measurements can be used to determine the kinetics of the different processes.

Further to the parameters discussed above and independent of process efficiencies related to support and reactor design configurations, obtaining intrinsic kinetics of the photocatalyst during the reaction is crucial for a true efficiency baseline and for comparison with other catalysts. This means decoupling the local rate‐dependence on light and mass transfer by operating the reaction under known, characterizable particle size and spatial distribution, agglomeration, temperature and reactant flow regime, and compositions. Systematically varying reactant and product compositions one at a time allows the reaction‐rate orders of reactants and products to be determined, necessary in the formulation of the rate law. The flow regime though the reactor bed can be characterized experimentally through a tracer study, in which a pulse or step of a suitable tracer fluid is injected while the concentration at the outlet is continuously measured in order to obtain a residence time distribution of the tracer. The residence time distribution can be fitted to parametric models that define how close in operation to an ideal plug flow or continuous stir tank reactor the photoreactor is. Similarly well‐known analytical models for ideal systems (allowing for small deviations) can be employed to solve the radiative transfer equation to obtain optical properties of the catalyst bed.^[^ ^76^, ^81^, ^83^ ^]^

## 6 A Way Forward

As is shown in the aforementioned work, each stage in the solar photon‐to‐product process has been investigated in some manner by machine learning (ML). However, due to the variety of parameters and analytic approaches to each process stage, it may not be possible to simply optimize each stage separately such that the solar‐to‐product figure of merit is maximized. One possibility is to use supervised ML as a powerful tool for making a variety of predictions for catalyst and reactor properties,^[^ ^84^, ^85^, ^86^, ^87^, ^88^, ^89^, ^90^ ^]^ where relevant features in catalyst and reactor design (i.e., electronegativity, band center, surface area, reaction conditions, and reactor geometry/topology) can serve as input features for training a model on desired outputs (i.e., product formation rate, selectivity, stability, and efficiency).^[^ ^91^, ^92^, ^93^ ^]^ Within photocatalysis, ML has been successfully employed, for example, to predict perovskite materials (using features including from electronegativity, light intensity, photocatalyst quantity, and calcination temperature)^[^ ^94^ ^]^ and layered double hydroxides (using elemental and structural features generated from external packages^[^ ^95^, ^96^ ^]^ and based on local chemical hardness) for water splitting, organic heterojunction photocatalysts (using electronic descriptors such as electron affinity and reorganization energy) for hydrogen production,^[^ ^97^ ^]^ and optimal reaction conditions (flow rate and reactor temperature) for the degradation of dyes.^[^ ^98^ ^]^ In addition, models have also been built based on literature data for establishing trends and connections for products, catalysts, and material properties for CO_2_ photocatalysis in both the gas and liquid phases.^[^ ^99^ ^]^

Traditionally, most ML models have been “data‐driven”, meaning that the connection between input and output is established based solely on statistical fitting of the dataset provided. With these data‐driven statistical models, there are often challenges with interpretability,^[^ ^100^, ^101^, ^102^ ^]^ in that it typically decreases when model accuracy increases. In addition, one can sometimes obtain physically unreasonable results.^[^ ^103^, ^104^, ^105^ ^]^ As a result, neural networks and deep learning methods, which can be highly accurate, often yield results that are poorly interpretable. Fortunately, recent developments in so‐called “physics‐based” deep learning offer opportunities to construct robust and physically meaningful models from which interpretable information can be extracted.^[^ ^98^, ^99^, ^100^, ^101^, ^102^, ^103^ ^]^

The original concept of integrating domain knowledge into the supervised training of deep neural networks began by taking advantage of deep neural networks’ ability to operate as function approximators,^[^ ^106^ ^]^ essentially using them to approximate equations that are difficult/impossible to solve analytically or even numerically. By leveraging the nature of evaluating derivatives in the backpropagation of neural networks and applying recent developments in automatic differentiation,^[^ ^107^ ^]^ one can build models that minimize statistical error while satisfying the relevant physical constraints. The physical constraints are often expressed as residuals against certain boundary conditions and specific forms of partial differential equations (PDEs), and together with the statistical error, are combined into one loss function to minimize during the training process, enabling the construction of a physics‐informed statistical model. This concept of physics‐informed neural networks was popularized by Raissi et al.,^[^ ^108^, ^109^ ^]^ and it has been increasingly applied across studies over the past several years.^[^ ^110^, ^111^, ^112^, ^113^, ^114^ ^]^ By combining a neural network with physically meaningful constraints, one can obtain a surrogate model for describing complex behavior. Importantly, these physics‐informed models can be trained on small data sets, are compatible with noisy and high‐dimensional data, and are quite capable at solving inverse problems, all of which are relevant to gas‐phase CO_2_ heterogeneous photocatalysis.

The original approach of physics‐based deep learning^[^ ^108^ ^]^ was primarily based on 1) inferring solutions to, and 2) finding a best fit for the values of parameters/constants that accompany a PDE, and it required information on the general forms of the equations and prior knowledge of the system at hand. In terms of catalysis, the production rate, selectivity, and yield of a desired product are among the most important metrics in evaluating performance. These metrics are almost always functions of the thermodynamic and kinetic attributes of the relevant system, but these thermodynamic and kinetic parameters depend heavily and in a very complex manner on the operating conditions (i.e., temperature, partial pressure, and flow rate), reactor type, and catalyst attributes (i.e., composition, morphology, structure, and type of support). The desired metrics are more like composite functions of these directly measurable and well‐defined quantities, and obtaining empirically a general form for the relevant equations is often not straightforward and can require significant expert knowledge and experience.

Thankfully, a variety of other methods have built on or expanded beyond the original approach to incorporate information on chemical kinetics,^[^ ^112^, ^115^, ^116^ ^]^ thermochemical/thermophysical information,^[^ ^115^, ^117^, ^118^, ^119^, ^120^ ^]^ mass/energy balances,^[^ ^121^, ^122^, ^123^ ^]^ reactor engineering dynamics,^[^ ^124^, ^125^ ^]^ computed interatomic potentials,^[^ ^126^ ^]^ and optics.^[^ ^127^ ^]^ Many of these expanded approaches enable embedding of chemical information and may no longer be restricted to only finding solutions to and/or fitting PDEs with a previously known form. We propose referring to these models as “physiochemically informed neural networks”. However, we should still keep in mind that they are essentially just neural networks trained on constraints (physical, chemical, and other considerations) beyond just the statistics of the data itself. As an example, so‐called “kinetics‐informed” neural networks, taking into consideration the types of species (i.e., gas substrate, surface, adsorbed substrate, and desorbed product) involved, have been generated to reproduce the results from microkinetic modeling.^[^ ^115^ ^]^ There are also other approaches involving neural ordinary differential equations (ODEs).^[^ ^128^, ^129^ ^]^ One of them incorporated constraints on material balance, reaction rates relative to equilibrium, the Arrhenius relationship, and the simple fact that the rate is 0 when no reagents are present, successfully producing a model from both simulated and experimental data.^[^ ^129^ ^]^

**Figure 7:**
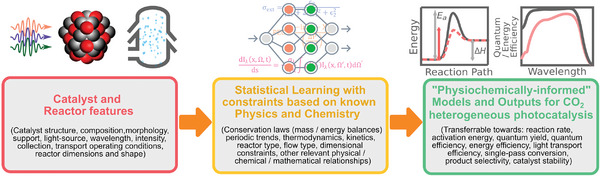
By training a model to ensure its outcomes satisfy the constraints of established physics and chemistry knowledge, one can use the relevant catalyst and reactor features to build physiochemically informed statistical models for predicting and optimizing various aspects of CO_2_/H_2_O heterogeneous photocatalysis.

To accelerate the development of gas‐phase CO_2_ photocatalysts and photoreactors, it may be most useful to consider together relevant target descriptors from the nanoscale (i.e., light absorption and activation energies) to the macroscale (i.e., product formation rate and selectivity) in one unified approach to achieve the best balance of outcomes (**Figure** **7**). This is despite the observation that quite often the best catalyst at the lab scale may not necessarily perform the best in an “optimal” reactor and vice versa, and also that a “research catalyst” can be quite different from a “technical catalyst”.^[^ ^130^ ^]^ Choosing the appropriate physiochemically informed models from the variety available in the literature and deciding how they can be applied in parallel will be a task that needs to be addressed. Using a specific model (i.e., the technique from Raissi et al. to solve and/or get the coefficients of the radiative transfer, mass transfer, and diffusion equations) can be useful toward addressing a specific question (in other words, a part of the bigger problem), but we may miss out on the connections and interactions that occur between different parts of the problem that may be important to consider (i.e., how the activity that arises from a certain surface structure and composition at the nanoscale may be affected by various reaction conditions and different types of reactor engineering). In many cases, to accurately describe the complex relationships within gas‐phase heterogeneous photocatalysis, development of a new physiochemically informed model may be necessary. Of note, the recently developed Bayesian Machine Scientist may be helpful in putting complex relationships into concrete and optimizable forms.^[^ ^131^ ^]^

Nevertheless, amid the ongoing effort of incorporating domain knowledge into statistical models, we would like to call for more development of these physiochemically informed models in the context of photocatalysis design and photoreactor engineering. Hopefully, with the aid of these new models, gas‐phase CO_2_ heterogeneous photocatalysis will become commercially relevant.

## 7 Big Picture Context

The Holy Grail of General Artificial Intelligence, fusing the speed and number‐crunching abilities of machines with the intuition of human minds, was thought by some to be impossible. However, the recent strides in Language Neural Networks such as Generative Pre‐Trained Transformer (GPT) (the large language model that powers ChatGPT) have led to renewed optimism that General AI is feasible within our generation. This also prompts the re‐evaluation of problems previously thought to be unsolvable due to their scale and complexity. In this article, we distill four pivotal design factors of CO_2_‐to‐solar fuel plants—some underserved by machine learning optimization—and discuss how Physics Neural Network can cut the Gordian Knot.

## Conflict of Interest

The authors declare no conflict of interest.

## Author Contributions

J.L. conceptualized the manuscript and prepared Section 2 on light distribution as well as performing paper edits. A.W. prepared Section 5 on physics neural networks. A.M. and A.G. prepared Section 4 on photoexcitation and material science. A.T. prepared Section 3 on catalyst support. A.T. prepared Section 1 on light collection and G.O. is the principal investigator.
